# Cerebrospinal fluid biomarkers for differentiation of frontotemporal lobar degeneration from Alzheimer's disease

**DOI:** 10.3389/fnagi.2013.00006

**Published:** 2013-02-21

**Authors:** David J. Irwin, John Q. Trojanowski, Murray Grossman

**Affiliations:** ^1^Department of Pathology and Laboratory Medicine, Center for Neurodegenerative Disease Research, Alzheimer's Disease Core Center, Institute on Aging, University of PennsylvaniaPhiladelphia, PA, USA; ^2^Department of Neurology, Center for Frontotemporal Dementia, Perelman School of Medicine, University of PennsylvaniaPhiladelphia, PA, USA

**Keywords:** cerebrospinal fluid, biomarker, tau, Aβ_1−42_, frontotemporal dementia, primary progressive aphasia, Alzheimer's disease

## Abstract

Accurate ante mortem diagnosis in frontotemporal lobar degeneration (FTLD) is crucial to the development and implementation of etiology-based therapies. Several neurodegenerative disease-associated proteins, including the major protein constituents of inclusions in Alzheimer's disease (AD) associated with amyloid-beta (Aβ_1−42_) plaque and tau neurofibrillary tangle pathology, can be measured in cerebrospinal fluid (CSF) for diagnostic applications. Comparative studies using autopsy-confirmed samples suggest that CSF total-tau (t-tau) and Aβ_1−42_ levels can accurately distinguish FTLD from AD, with a high t-tau to Aβ_1−42_ ratio diagnostic of AD; however, there is also an urgent need for FTLD-specific biomarkers. These analytes will require validation in large autopsy-confirmed cohorts and face challenges of standardization of within- and between-laboratory sources of error. In addition, CSF biomarkers with prognostic utility and longitudinal study of CSF biomarker levels over the course of disease are also needed. Current goals in the field include identification of analytes that are easily and reliably measured and can be used alone or in a multi-modal approach to provide an accurate prediction of underlying neuropathology for use in clinical trials of disease modifying treatments in FTLD. To achieve these goals it will be of the utmost importance to view neurodegenerative disease, including FTLD, as a clinicopathological entity, rather than exclusively a clinical syndrome.

## Introduction

Most neurodegenerative diseases are characterized by specific abnormally-modified protein aggregates, with resulting neuronal cell loss and gliosis. The gold standard for diagnosis is microscopic examination at autopsy; however, there is considerable variability of clinical manifestations associated with underlying neuropathological diagnoses, as clinical symptoms most often reflect the regional burden of pathology within the central nervous system (CNS) rather than the specific underlying proteinopathy. This is especially true in the heterogeneous family of frontotemporal lobar degeneration (FTLD) clinical syndromes.

**Figure 1:**
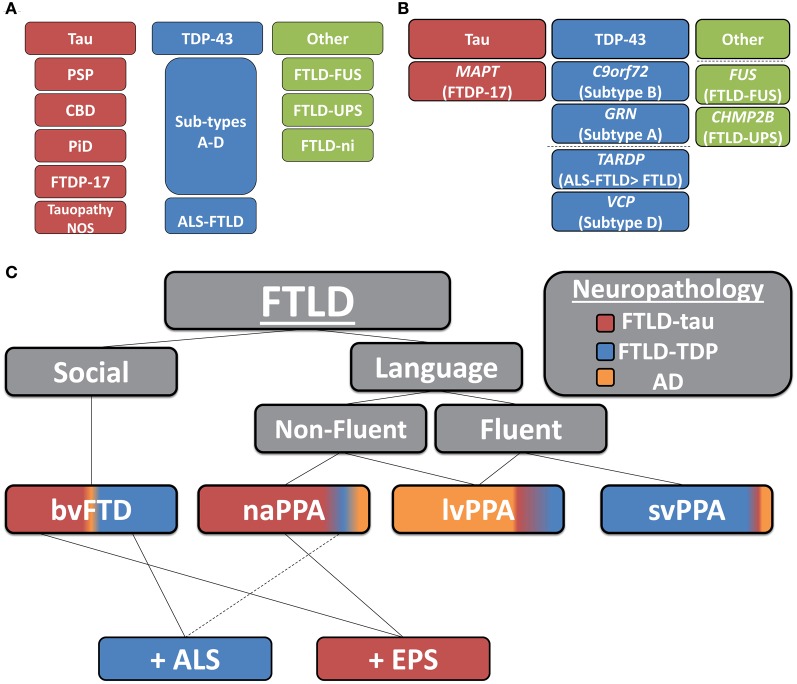
**Clinicopathological and genetic associations in FTLD. (A)** Neuropathological classification of FTLD-tau and FTLD-TDP subtypes (PSP, progressive supranuclear palsy; CBD, corticobasal degeneration; PiD, Pick's disease; FTDP17, frontotemporal dementia with Parkinsonism linked to chromosome 17; Tauopathy NOS, unclassifiable tauopathy; Subtypes A–D, morphological subtypes of FTLD-TDP; ALS-FTLD, amyotrophic lateral sclerosis with FTLD-TDP; FTLD-FUS, FTLD with fused in sarcoma protein inclusions; FTLD-UPS, FTLD with tau- and TDP-43-negative ubiquitinated inclusions; FTLD-ni, FTLD in the absence of significant neuropathological inclusions), **(B)** pathogenic mutation associations with underlying neuropathology (dashed-line separates less common molecular etiologies of FTLD; *MAPT*, tau resulting in FTDP-17; *C90rf72*, pathogenic hexanucleotide expansion resulting in FTLD and/or ALS associated with FTLD-TDP B; *GRN*, progranulin resulting in FTLD-TDP type A; *TARDP*, TDP-43 resulting in ALS ± FTLD and less commonly FTLD; *VCP*, valosin-containing protein resulting in inclusion body myopathy with Paget's disease of bone and frontotemporal dementia with FTLD-TDP subtype D; *FUS*, fused-in sarcoma protein resulting in FTLD-FUS; and *CHMP2B*, charged mutlivesciular body protein 2B resulting in FTLD-UPS), **(C)** clinicopathological correlations of FTLD (colored regions of clinical syndromes represent relative percentages of neuropathological subtypes found in autopsy studies; AD, Alzheimer's disease; bvFTD, behavioral variant of FTLD; PPA, primary progressive aphasia; svPPA, semantic variant PPA; naPPA, nonfluent agrammatic variant PPA; lvPPA, logopenic variant PPA; +ALS, co-morbid amyotrophic lateral sclerosis; +EPS, co-morbid extra-pyramidal Parkinsonian symptoms: i.e., features of akinetic-rigid syndromes of PSP or corticobasal syndrome).

**Figure 2:**
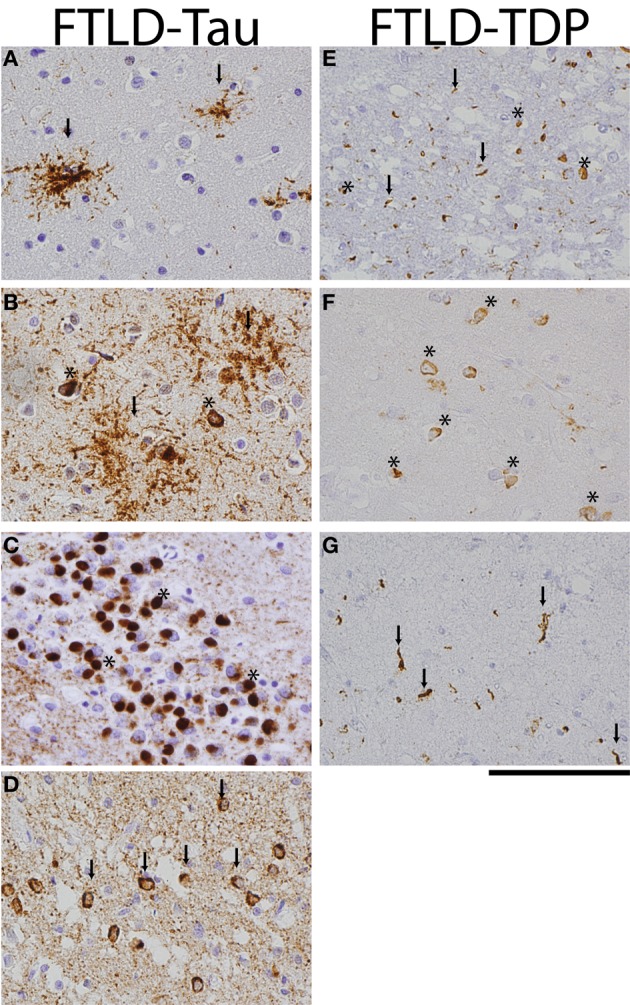
**FTLD-Tau and FTLD-TDP histology.** Photomicrographs of FTLD-tau **(A–D)** and FTLD-TDP **(E–G)** visualized with immunohistochemistry (PHF-1 and pTDP 409/410 for tau and TDP, respectively). **(A)** PSP frontal cortex with tau-positive tufted astrocytes (arrows), **(B)** CBD temporal cortex with diffuse astrocytic plaques (arrows) and neuronal tangles (asterisks), **(C)** Pick's disease with round tau-positive Pick bodies (asterisks) in the dentate nucleus of the hippocampus, **(D)** FTDP-17 case with p.P301L pathogenic mutation with tau-positive neuronal tangles (arrows) and diffuse neuropil threads in temporal cortex, **(E)** FTLD-TDP subtype A with cytoplasmic neuronal inclusions (asterisks) and short dystrophic neurites (arrows) in superficial layers of frontal cortex, **(F)** FTLD-TDP subtype B with prominent cytoplasmic inclusions (asterisks) in deep temporal cortical layer, and **(G)** long dystrophic neurites (arrows) in superficial layers of mid-frontal cortex of a patient with FTLD-TDP subtype C. Scale bar = 100 μm.

Two main pathologic FTLD subtypes exist (Figures 1A, 2): cases with inclusions formed from the microtubule-binding protein tau (FTLD-tau) and those with TAR DNA binding protein-43 (TDP-43) pathology (FTLD-TDP) (Mackenzie et al., 2010). FTLD-tau includes the following tauopathies (Figures 2A–D): Pick's disease (PiD), corticobasal degeneration (CBD), progressive supranuclear palsy (PSP), FTD and parkinsonism linked to chromosome 17 (pathogenic *MAPT* mutations; FTDP-17), and unclassifiable tauopathies (Mackenzie et al., 2010). FTLD-TDP (Figures 2E–G) can be subdivided into four subtypes (A–D) based on the morphology and distribution of lesions (Mackenzie et al., 2011) and can also be associated with TDP-43 inclusions in the anterior horn of the spinal cord and gliosis of the corticospinal tracts, suggesting a continuum of FTLD with amyotrophic lateral sclerosis (ALS; FTLD-ALS) (Geser et al., 2008, 2009). A smaller number of FTLD cases are associated with inclusions of another DNA-binding protein, fused-in-sarcoma protein (*FUS;* FTLD-FUS), or other rare, less-defined pathologies (FTLD-UPS, FTLD-ni) (Mackenzie et al., 2010). The major genetic etiologies resulting in FTLD are exclusively associated with specific underlying neuropathologies (Figure 1B), despite heterogeneous expression of FTLD clinical syndromes, and include pathogenic mutations in the gene for progranulin (*GRN*) (Baker et al., 2006; Cruts et al., 2006), tau (*MAPT*) (Hutton et al., 1998), and C9orf72 (*C9orf72)* (Dejesus-Hernandez et al., 2011; Renton et al., 2011). Less common genetic etiologies of FTLD include: valosin-containing protein (*VCP*) resulting in inclusion body myopathy with Paget's disease of bone and frontotemporal dementia with FTLD-TDP subtype D neuropathology, *TARDBP* coding for TDP-43 protein and causing ALS or ALS-FTLD (rarely FTLD-TDP alone), *CHMP2B* coding for charged mutlivesciular body protein 2B and resulting in FTLD-UPS, and mutations in *FUS* causing FTLD-FUS (Figure 1B) (Mackenzie et al., 2010).

Clinically, FTLD can be broadly divided into two main subtypes, those with predominant behavioral and social comportment disorder (behavioral-variant frontotemporal dementia, bvFTD) (Rascovsky et al., 2011) and those with primary language disturbances (primary progressive aphasia, PPA) (Mesulam, 1982, 2001). Among PPA patients, three subgroups have been recently divided (Gorno-Tempini et al., 2011) into the logopenic (lvPPA) (Gorno-Tempini et al., 2004, 2008), semantic (svPPA) (Hodges and Patterson, 2007), and non-fluent aggramatic variant (naPPA) (Turner et al., 1996). Clinicopathological correlations of these syndromes are complex (Josephs, 2008; Grossman, 2010). For example, large studies of autopsy-confirmed FTLD (behavioral and aphasic variants) find roughly equal numbers of FTLD-tau and FTLD-TDP (Hodges et al., 2004; Kertesz et al., 2005; Knopman et al., 2005; Shi et al., 2005; Forman et al., 2006). Furthermore, a primary neuropathological diagnosis of Alzheimer's disease (AD) has been found in up to 30% of autopsy-confirmed clinically defined FTLD cohorts (Kertesz et al., 2005; Knopman et al., 2005; Forman et al., 2006; Knibb et al., 2006). Examination of focal presentations of AD found it to be the primary diagnosis in 7% of bvFTD, 44% of naPPA, 10% of svPPA, and 50% of the extrapyramidal and cognitive disorder, corticobasal syndrome (CBS) patients (Alladi et al., 2007). Others have also found a substantial proportion of AD in PPA cases (Forman et al., 2006; Knibb et al., 2006) especially in lvPPA (Grossman et al., 2008; Mesulam et al., 2008; Grossman, 2010) and also CBS (Lee et al., 2011). Thus, differentiation of AD and FTLD spectrum disorders poses a serious diagnostic challenge for clinicians.

Within the FTLD neuropathological spectrum, examination of the specific clinical subtypes finds varying degrees of association with FTLD-tau and FTLD-TDP (Figure 1C). FTLD-tau has been overrepresented in some naPPA cohorts (Hodges et al., 2004; Josephs et al., 2006a,b; Knibb et al., 2006; Snowden et al., 2007; Mesulam et al., 2008; Grossman et al., 2012), especially when associated with apraxia of speech (Josephs et al., 2006a; Snowden et al., 2007) and svPPA has been predominantly associated with TDP-43 pathology (Hodges et al., 2004; Josephs et al., 2006a; Snowden et al., 2007; Grossman et al., 2008); while bvFTD contains similar proportions of FTLD-tau and FTLD-TDP (Forman et al., 2006; Josephs et al., 2006b; Snowden et al., 2007). Extrapyramidal symptoms may predict a tauopathy (Forman et al., 2006; Josephs et al., 2006b) while co-morbid ALS is almost certainly due to TDP-43 aggregation (Shi et al., 2005; Forman et al., 2006; Josephs et al., 2006b). Clinicopathological associations from these large autopsy studies are summarized in Figure 1C.

A major challenge in the development and implementation of disease-modifying therapy in FTLD is the accurate identification of the neuropathological diagnosis during life, including differentiation from AD, so that patients may be triaged to the appropriate protein-targeted therapy (i.e., tau or TDP-43 targeted agents).

Biofluid biomarkers have the potential to optimize diagnostic accuracy and detect disease earlier in the course of an illness and possibly pre-symptomatically, such as prior to structural changes of neurodegeneration seen on neuroimaging (Hu et al., 2010a; Jack et al., 2010), making further exploration in this area promising for the development of disease modifying treatments. In addition, some clinical measures of disease progression in FTLD, including functional scales, may be limited by floor- and ceiling-effects (Knopman et al., 2008), so biofluid biomarkers are potentially attractive surrogate end points for use in clinical trials (Boxer et al., 2012b). The cerebrospinal fluid (CSF) is relatively easy to obtain and contains a direct connection to the pathological milieu in central nervous system, making it a desirable biofluid for study. In this review we will discuss the current state of CSF biomarker research in FTLD in terms of differentiation from AD and future directions and challenges for the field in development of FTLD-specific biomarkers.

## Alzheimer's disease related csf biomarkers: Aβ_1−42_ and tau

### Studies in alzheimer's disease

As a first step in biofluid-based biomarker assessment of neurodegenerative disease, it is valuable to distinguish broadly between AD and FTLD. CSF values of the major constituents of AD pathology, tau and β-amyloid, (Aβ_1−42_) have been widely studied using immune-based analytical platforms in AD and amnestic mild cognitive impairment (MCI) patients, with lower Aβ_1−42_ values and higher levels of total- and phosphorylated-tau (t-tau, p-tau) compared with controls across multiple large studies (Shaw et al., 2009, 2011; De Meyer et al., 2010; Trojanowski et al., 2010; Weiner et al., 2010). Furthermore, our group has shown prognostic utility of these markers by accurately predicting MCI conversion to AD (Shaw et al., 2009; De Meyer et al., 2010).

The majority of atypical clinical presentations of AD in early-onset patients consisting of predominantly visuo-spatial difficulties (i.e., consistent with poster cortical atrophy) or asymmetric apraxia/rigidity (i.e., consistent with CBS) may have a similar CSF biomarker profile to that of typical amnestic-AD (De Souza et al., 2011; Seguin et al., 2011), with a further elevated t-tau level in one study (Koric et al., 2010). Elevated CSF t-tau and low Aβ_1−42_ levels have also been described in some PPA patients (i.e., lvPPA) (Bibl et al., 2011; De Souza et al., 2011) most likely due to underlying AD neuropathology in these individuals; however, to our knowledge no autopsy-confirmed studies of atypical clinical AD presentations have been performed.

The exact relationship between AD neuropathologic change (i.e., tau neurofibrillary pathology and Aβ_1−42_ extracellular plaques) and observed measurement of these analytes in CSF is unclear; however, the total tau level is thought to reflect underlying neurodegeneration and neuron loss, as elevations are also seen in other CNS insults (Otto et al., 1997; Hesse et al., 2000; Jin et al., 2006; Ost et al., 2006; Krut et al., 2013). Lower Aβ_1−42_ CSF levels may be the result of sequestration of soluble interstitial brain Aβ_1−42_ into extracellular plaques as there is an inverse correlation of CSF Aβ_1−42_ levels and the degree of cortical plaque pathology (Tapiola et al., 2009; Patel et al., 2012; Seppala et al., 2012) and *in vivo* neuroimaging evidence of amyloidosis (Fagan et al., 2006). Phosphorylated epitopes of tau (p-tau) can be measured in CSF as well; while most phospho-epitopes of tau are also found in healthy non-diseased brains and are not AD-specific, pathological tau species overall are highly phosphorylated in AD (Matsuo et al., 1994) and this altered state reflects the elevated levels of p-tau seen in AD. The most commonly studied p-tau epitopes are serine 181 (p-tau_181_) (Vanmechelen et al., 2000), and threonine 231 (p-tau_231_) (Buerger et al., 2002a,b).

### Studies in frontotemporal lobar degeneration

FTLD is not characterized pathologically by cerebral Aβ_1−42_ amyloidosis, and only FTLD-tau is characterized by significant tau inclusions. From this perspective, measures of CSF t-tau and Aβ_1−42_ may have helpful diagnostic utility in excluding AD neuropathology. Indeed, in clinically-defined cohorts AD cases have higher levels of t-tau, p-tau_181_ and lower levels of Aβ_1−42_ compared to FTLD and controls in group-wise comparisons (Blennow et al., 1995; Arai et al., 1997; Green et al., 1999; Sjogren et al., 2000a, 2001; Vanmechelen et al., 2000; Riemenschneider et al., 2002; Clark et al., 2003; Pijnenburg et al., 2004, 2007; Schoonenboom et al., 2004, 2012; Engelborghs et al., 2006; Bibl et al., 2007, 2011; Kapaki et al., 2008; Verwey et al., 2010; De Souza et al., 2011; Gabelle et al., 2011; Van Harten et al., 2011).

A major challenge in FTLD CSF biomarker studies is the heterogeneity of the condition (Figure 1), making autopsy-confirmation of diagnostic classification a crucial issue. As mentioned previously, up to 30% of clinically-defined FTLD cohorts may have underlying AD neuropathologic change as the etiology of their symptoms (Kertesz et al., 2005; Knopman et al., 2005; Forman et al., 2006; Knibb et al., 2006) and contamination with these atypical AD cases could influence results significantly. Indeed, examination of diagnostic accuracy of CSF t-tau and Aβ_1−42_ in a large autopsy-confirmed dementia cohort found that use of the clinical diagnosis, rather than neuropathological diagnosis as the gold standard for biomarker performance resulted in a 10–20% underestimation of biomarker accuracy (Toledo et al., 2012). Furthermore, since 1995 there has been over a 10-fold increase in the number of FTLD manuscripts published (NLM/NIH, 2012) and due to this exponential increase in research in the field and our expanding knowledge of FTLD, clinical criteria (Gorno-Tempini et al., 2011; Rascovsky et al., 2011) have evolved resulting in refinement of our clinical definitions. Indeed, the emergence of the new clinical variant of PPA, lvPPA (Gorno-Tempini et al., 2008, 2011), which is most often associated with AD neuropathology (Mesulam et al., 2008; Rabinovici et al., 2008; Grossman, 2010) (Figure 1C), and therefore suggested to be excluded from FTLD clinical trials (Knopman et al., 2008), could influence group-wise CSF tau and Aβ_1−42_ results. Thus, the makeup of clinical cohorts used in earlier studies may not be entirely translatable to newer studies, limiting the meaningful interpretation of the literature of clinically-derived cohorts.

As such, study of autopsy/genetic-confirmed cases has been a focus for our center. In an early study of autopsy-confirmed cases by our group, AD was differentiated from a mixed dementia cohort (including 13 FTLD cases) with reasonable sensitivity (72%) and specificity (69%) using CSF t-tau levels (Clark et al., 2003). Focused analysis of FTLD (with autopsy confirmation in 9 cases) in a later study found lower levels of t-tau and higher levels of Aβ_1−42_ than AD, and roughly 30% of FTLD cases had significantly decreased t-tau from controls (Grossman et al., 2005). In a follow-up large autopsy/genetically confirmed FTLD series (*n* = 30) t-tau levels were significantly lower in FTLD than AD, while similar to controls on group-wise comparison; individual-case analysis revealed that a considerable subset of FTLD patients had markedly low t-tau values (Bian et al., 2008). Interestingly, FTLD cases with substantially lower t-tau levels included both FTLD-tau and FTLD-TDP (Bian et al., 2008), although a non-significant trend was found for lower t-tau in FTLD-tau (Hu et al., 2011). Furthermore, FTLD was differentiated from AD with high accuracy using the t-tau/Aβ_1−42_ ratio; that is, FTLD cases had a lower ratio (lower t-tau and higher Aβ_1−42_) (Bian et al., 2008).

Measurement of these analytes in the CSF in most studies utilizes one of two immune-based platforms: enzyme-linked immunosorbent assay (ELISA; Innotest, Innogenetics), and a multiplex assay based on flow-cytometry of antibody-coated fluorescent beads (INNO-BIA AlzBio3 xMAP; Luminex, Innogenetics). Absolute values obtained from these platforms differ because the coefficient of variance (%CV) with the xMAP Luminex platform is much narrower than with ELISA, but they are highly correlated (Olsson et al., 2005; Lewczuk et al., 2009; Fagan et al., 2011; Wang et al., 2012) and have similar levels of diagnostic accuracy for AD (Fagan et al., 2011; Wang et al., 2012) and differentiating AD from FTLD (Toledo et al., 2012). Thus, values from one platform can be effectively transformed into equivalent units of the other using a conversion factor (Fagan et al., 2011) Indeed, we were able to transform values obtained from ELISA to equivalent xMAP units using linear regression to create a larger autopsy/genetic-confirmed FTLD dataset and help confirm our pervious observations of the diagnostic utility of the t-tau/Aβ_1−42_ ratio to differentiate FTLD from AD (Irwin et al., 2012b). Maximizing available data is crucial for these extremely valuable and well-annotated research samples. In summary, in multiple large-scale autopsy-confirmed studies we have demonstrated the diagnostic utility of CSF t-tau, p-tau, and Aβ_1−42_ in differentiation of AD and FTLD (Bian et al., 2008; Irwin et al., 2012b; Toledo et al., 2012).

Few other CSF studies have used autopsy-confirmed cohorts of FTLD patients (Table 1). One study included 10 autopsy-confirmed FTLD patients and found similar results of lower t-tau and p-tau_181_ levels in FTLD compared with AD, with high diagnostic accuracy of p-tau_181_ (Koopman et al., 2009). Another study including 12 confirmed FTLD patients described “slightly elevated tau levels” in several patients compared to an age-dependent reference range and low compared to the majority of AD cases (Brunnstrom et al., 2010). Neuropathological subgroups of FTLD (FTLD-TDP, *n* = 5 and FTLD-tau, *n* = 7) had similar mean values, with 4/12 patients below the reference limit by >70 pg/ml (Brunnstrom et al., 2010). Thus, this study also found a subset of individual FTLD patients with lower than normal t-tau levels. The diagnostic utility of t-tau/Aβ_1−42_ in differentiating FTLD was not systematically explored in this small group of AD cases (*n* = 8). Finally, to our knowledge the only additional studies utilizing autopsy-confirmed FTLD cohorts included a small number of FTLD cases (<10) in a non-AD category, with no direct comparison of FTLD and AD (Engelborghs et al., 2008; Tapiola et al., 2009; Schoonenboom et al., 2012). Thus, further study is required in large prospective, autopsy-confirmed samples to confirm our observations.

**Table 1:** **Comparative studies of CSF biomarkers in autopsy/genetic-confirmed FTLD and AD cohorts**.

**Study**	**Patients**	**Aβ_1−42_**	**t-tau**	**p-tau_181_**	**Diagnostic accuracy (AD vs. FTLD)**
Clark et al., 2003	(10) FTLD(74) AD^*^73(4) CN	AD < FTLD, CN	CN < FTLD < AD	NA	No statistical analysis of FTLD diagnostic accuracy performed
Grossman et al., 2005	73 (11) FTLD(17) AD13 CN	AD < FTLD, CN	CN, FTLD < AD	CN, FTLD < AD	t-tau
					AUC = 0.86, sens = 74%, spec = 82.4%
Bian et al., 2008	(30) FTLD(19) AD13 CN	AD < FTLD, CN	CN, FTLD < AD	NA	t-tau/Aβ_1−42_
					AUC = 0.93, sens = 78.9%, spec = 96.6%
Engelborghs et al., 2008	(2) FTLD(73) AD^*^100 CN	NA	NA	NA	No statistical analysis of FTLD diagnostic accuracy performed
Koopman et al., 2009	(10) FTLD(95) AD	AD < FTLD	FTLD < AD	FTLD < AD	p-tau_181_
					AUC = 0.85, sens = 91%, spec = 80%
Tapiola et al., 2009	(9) FTLD(83) AD	NA	NA	NA	No statistical analysis of FTLD diagnostic accuracy performed
Brunnstrom et al., 2010	(12) FTLD(8) AD^*^	NA	NA	NA	No statistical analysis of FTLD diagnostic accuracy performed
Irwin et al., 2012b	(20) FTLD(41) AD^*^	NA	NA	NA	t-tau/Aβ_1−42_
					AUC = 0.99, sens = 90–100%, spec = 90–96%
Toledo et al., 2012	(71) AD(29) FTLD66 CN	AD < FTLD < CN	CN, FTLD < AD	CN, FTLD < AD	t-tau/Aβ_1−42_ (ELISA)
					AUC = 0.96, sens = 90, spec = 82%
					p-tau_181_/Aβ_1−42_ (xMAP)
					AUC = 0.98, sens = 100%, spec = 88%

*Other diagnostic groups that may be present in some studies are omitted and only direct comparisons of FTLD group to AD or CN are reported. “<” or “>” denotes significant difference between groups and “,” denotes non-significant difference between groups, () denotes autopsy/genetic confirmed cohort*.

CN, non-demented controls;

* *, AD group contains cases with co-morbid Lewy Body or Vascular Disease; NA, Not assessed; AUC, Area under the curve for receiver operating curve analysis; ELISA, enzyme-linked immunosorbent assay; xMAP, luminex multiplex assay*.

The higher Aβ_1−42_ in FTLD compared to AD most likely reflects the absence of significant cerebral amyloidosis while the biological basis for observed low CSF t-tau in some FTLD patients is uncertain. One possibility is related to cortical tau depletion (Zhukareva et al., 2001, 2003; Grossman et al., 2005) through sequestration into the neuronal and glial inclusions in the absence of significant extracellular tau pathology (FTLD-tau) Dickson, 2004, such as extracellular “ghost tangles” as seen in AD (Schmidt et al., 1988), or altered post-translational stability of tau in FTLD-TDP (Zhukareva et al., 2001, 2003); furthermore, CSF t-tau does appear related to underlying FTLD pathophysiology as t-tau levels in FTLD patients correlated to areas of frontal and temporal cortical atrophy on magnetic resonance imaging (MRI) (Grossman et al., 2005; McMillan et al., 2013). Further study of CSF protein dynamics in animal models of disease may help clarify these seemingly discordant associations of low tau levels with underlying neuropathology in FTLD-tau and FTLD-TDP.

Despite the clear distinction of t-tau and Aβ_1−42_ levels between AD and FTLD, there is more variability in the literature for the relationship of these markers in FTLD compared with non-demented controls (Table 1). There are several reasons for these discrepancies; first, even in most autopsy-based studies, autopsy data on controls is lacking (Table 1) and a significant proportion of non-demented elderly can have underlying AD neuropathology (Davis et al., 1999), and thus influence CSF analyte measures. Next, even with pathologic confirmation, patient classification in FTLD is challenging, as another potential confounding issue is the presence of mixed pathologies in dementia patients. Indeed, our group has shown in a large autopsy-confirmed sample that mixed pathology is present in roughly 30% of cases, and that FTLD patients with significant AD neuropathologic change can influence the CSF t-tau and Aβ_1−42_ levels, causing higher t-tau and lower Aβ_1−42_ in cases with mixed FTLD and AD pathology compared to “pure” FTLD (Toledo et al., 2012). Additionally, a recent largely clinically-defined cohort study found an AD CSF biomarker profile in 30% of FTLD (Schoonenboom et al., 2012) which may be due, in part, to mixed pathology or inclusion of atypical AD cases mimicking the FTLD clinical syndrome (Toledo et al., 2012). Thus, the use of autopsy-confirmed samples is essential for in-depth study and validation of the diagnostic accuracy of potential biomarkers in FTLD.

Finally, variability in measurement between studies is another potential issue as significant variation between centers in absolute values measured in “spiked” pooled CSF control samples with known concentrations of analyte has been described (Shaw et al., 2011). These discrepancies are most likely due to sources of variation in CSF collection, handling and storage (pre-analytical), equipment, reagents and methods of analysis (analytical), and data management and interpretation (post-analytical) (Mattsson et al., 2011). For these reasons, large scale studies of measurement precision of these analytes and coordinated multi-center quality control programs with standard operating procedures to minimize these sources of variation have been conducted (Mattsson et al., 2011; Shaw et al., 2011).

Despite these issues, we have demonstrated (Bian et al., 2008; Irwin et al., 2012b; Toledo et al., 2012) that these AD-specific analytes (t-tau to Aβ_1−42_ ratio) may perform within the range of sensitivity and specificity (>80%) for use in clinical trials (Trojanowski and Growdon, 1998) to differentiate FTLD from AD; however, these analytes are not as effective for differentiation of FTLD from normal controls (Bian and Grossman, 2007; Toledo et al., 2012). Although patients may present with decompensated psychiatric issues or other non-progressive non-degenerative etiologies resembling FTLD (phenocopy syndrome) (Kipps et al., 2010), these patients may be identified with serial clinical exams and neuroimaging (Kipps et al., 2010). The more urgent need is for FTLD-specific biomarkers and those that can differentiate between the two major neuropathologic subtypes (FTLD-tau and FTLD-TDP) (Hu et al., 2011).

## Future directions

### Further study of CSF tau and Aβ_1−42_

Previous work in large cross-sectional studies in AD suggests a temporal progression of dynamic biomarker change in AD (Jack et al., 2010, 2012), as Aβ_1−42_ amyloidosis, and resultant lower CSF Aβ_1−42_, is thought to occur decades before clinical symptoms emerge in AD, while increased CSF t-tau is thought to be a later event in disease progression and correlates more closely with cognitive decline. It is likely that t-tau, p-tau and potential novel CSF biomarkers could display similar changes throughout the course of disease in FTLD and could correlate with clinical symptoms. Few studies have examined the change in CSF biomarkers over time or their relation to clinical symptoms. One study included a follow up CSF analysis in one FTLD-tau patient, with similar t-tau and Aβ_1−42_, roughly 18 months between CSF collections (Brunnstrom et al., 2010). Interestingly, a recent study of bvFTD patients found a significant correlation with Aβ_1−42_ levels and cognitive performance, even after removal of patients with CSF profile suggestive of AD neuropathology (Koedam et al., 2012). These results could suggest an influence of co-morbid AD neuropathology; however autopsy information in these cases was lacking. Other studies in clinical series without autopsy confirmation found no association of these markers and clinical measures or disease severity (Riemenschneider et al., 2002; Engelborghs et al., 2006; De Souza et al., 2011). Further study of clinical correlates of CSF biomarkers and longitudinal profiles of CSF analyte change throughout the course of disease will be helpful.

Similar to the dominantly-inherited AD network (DIAN) initiative to study patients with known pathogenic mutations to cause AD (Bateman et al., 2012), study of prodromal FTLD patients with pathogenic mutations may provide additional insights into the temporal sequence of biomarkers in FTLD (Boxer et al., 2012a). Furthermore, CSF analyte levels in symptomatic patients with genetic forms of FTLD have not been explored in detail and could potentially differ from sporadic cases. Indeed, we found a more rapid rate of progression in cognitive measures corresponding to more severe neurodegeneration in *C9orf72*-associated FTLD (Irwin et al., 2013) and others have described unique neuroimaging patterns of atrophy across different genetic forms of FTLD (Whitwell et al., 2012). This evidence of biologic differences in genetic and sporadic FTLD suggest alterations in CSF biomarker profiles are also a possibility, although one study found similar levels of CSF tau and Aβ_1−42_ in genetically-confirmed FTDP-17 (*n* = 9) compared to sporadic FTLD (*n* = 17) (Rosso et al., 2003).

### Development of FTLD-specific biomarkers

In the context of disease-modifying therapies targeting a specific histopathologic abnormality, an important goal is to distinguish between FTLD due to TDP-43 and FTLD due to tau. Exploratory analyses for novel biomarkers that have diagnostic utility in FTLD are ongoing and include several basic approaches. First, measurement of biologically relevant molecules is the most straightforward approach, as tau and Aβ_1−42_ have been successful biomarker candidates in AD. Using this rationale, the two most obvious candidates for FTLD-specific biomarkers are TDP-43 progranulin. Indeed, TDP-43 has been detected in human CSF (Steinacker et al., 2008; Kasai et al., 2009) and serum (Foulds et al., 2008), suggesting elevated levels may occur in some patients with TDP-43 proteinopathies, but initial studies show limited diagnostic accuracy. Low serum progranulin may identify FTLD patients with a pathogenic *GRN* mutation resulting in progranulin haploinsufficiency (Ghidoni et al., 2008), which could be useful in monitoring potential progranulin-replacing therapies in development for FTLD (Boxer et al., 2012b).

Other biologically relevant potential biomarkers for FTLD include specific isoforms or neoepitopes of tau. Tau undergoes multiple post-translational modifications thought to contribute to tangle formation. Indeed, we found acetylation of tau at a specific residue in the microtubule-binding domain (MTBD) to be exclusively found in tauopathies, providing promise for this epitope as a useful marker of AD and FTLD-tau (Cohen et al., 2011; Irwin et al., 2012a). Translating these immunohistochemical observations to clinical assays may prove difficult, as levels of tau in CSF are near the lower limits of biologic detection (Hampel et al., 2010) limiting the further identification of a specific subset of tau in the form of a neoepitope; although one group has found promising evidence for diagnostic utility of specific C-truncated isoforms of tau in PSP through immunoprecipitation and western blotting techniques (Borroni et al., 2008, 2009) and others have developed assays to measure 3- and 4R tau in CSF (Luk et al., 2012). Alternatively-truncated forms of Aβ_1−42_ may also have diagnostic importance in FTLD (Pijnenburg et al., 2007; Bibl et al., 2011, 2012; Gabelle et al., 2011) and cytoskeletal proteins, such as neurofilament have also been explored (Sjogren et al., 2000b; De Jong et al., 2007). These potential biomarkers warrant further study and validation.

Another, possible approach is to screen a large number of potential analytes without an a priori biologic rationale in a proteomic analysis of CSF in FTLD. Indeed, using an immune-based multiplex approach our group found promising CSF biomarker candidates to differentiate FTLD-TDP and FTLD-tau with high sensitivity and specificity, but these candidate analytes need further study to confirm their utility as FTLD biomarkers (Hu et al., 2010b). Finally, other non-immune based methods, such as mass-spectrometry are also being explored to identify novel biofluid biomarkers in FTLD (Mattsson et al., 2008).

Potential FTLD-specific biofluid biomarkers will be faced with the same challenges of testing reliability and sources of variation (i.e., analytical, pre-/post-analytical) currently experienced by CSF t-tau and Aβ_1−42_ measurements. As such, coordinated and cooperative efforts between multiple centers will undoubtedly be necessary to help validate potential FTLD-specific CSF biomarkers prior to clinical use.

Most likely, a multimodal assessment incorporating potential novel biofluid biomarker values with clinical, neuroimaging and genetic markers may be the most effective approach to accurately identify FTLD subtypes. Neuropsychological testing can help differentiate AD from FTLD (Rascovsky et al., 2008; Libon et al., 2011) as routine cognitive measures may not be sensitive enough to detect the behavioral and language deficits in FTLD. Indeed, our group has explored quantitative approaches to language (Ash et al., 2006, 2009; Gunawardena et al., 2010) and social cognition (Massimo et al., 2009, 2013; Grossman et al., 2010; Eslinger et al., 2012; McMillan et al., 2012b) to examine brain-behavior relationships and improve diagnostic accuracy in FTLD. Neuroimaging is another potential method with diagnostic utility alone, or as an adjunct to clinical and biofluid biomarkers in FTLD; we have found combining neuropsychological testing and MRI can improve diagnostic accuracy in PPA (Hu et al., 2010c); and others find combination of CSF tau isoform levels and midbrain atrophy improve identification of PSP (Borroni et al., 2010). Multiple modalities of MRI methods, including diffusion-tensor imaging (DTI) of white matter may help identify FTLD patients in dementia cohorts. We have demonstrated increased diagnostic sensitivity to differentiate AD from FTLD cases using a combination of gray matter (GM) density and DTI measures (McMillan et al., 2012a). In addition, we have also discovered promising diagnostic utility for differentiating FTLD-tau and FTLD-TDP using DTI (unpublished data). Cortical atrophy and CSF biomarker levels appear to be highly correlated as we have recently demonstrated that GM density could predict CSF t-tau and Aβ_1−42_ levels, and these predicted values could accurately distinguish AD and FTLD (McMillan et al., 2013). These results indicate that MRI could potentially serve as a surrogate for CSF, which would have significant utility for patients where lumbar puncture would be difficult or for clinical trial endpoints where repeated lumbar puncture may be needed. Finally, recent genome-wide association studies (GWAS) have found risk alleles associated with FTLD-TDP (Van Deerlin et al., 2010) and FTLD-tau (Hoglinger et al., 2011). Further knowledge of clinical, neuroimaging, and biofluid correlates of these risk alleles in FTLD could provide further useful diagnostic and prognostic information. Thus, comparative studies of clinical, genetic, biofluid, and neuroimaging biomarkers in longitudinally followed, well-annotated, autopsy-confirmed subjects will be a powerful method for improving our understanding of the pathophysiology of FTLD and further directing diagnostic and treatment efforts.

## Summary

CSF measurements of Aβ_1−42_, t-tau, and p-tau in FTLD differ significantly from the abnormal levels seen in AD, and in a subset of both FTLD-tau and FTLD-TDP there are extremely low levels of t-tau of unclear etiology. These properties allow for accurate distinction of FTLD from AD in autopsy-confirmed cohorts, while FTLD-specific markers are still lacking.

As we move toward therapies that impact the progression of the disease and target the underlying pathophysiology in FTLD and other neurodegenerative disorders it will be essential for clinicians to view these disorders as clinicopathological entities with the underlying neuropathological substrate in mind. Indeed, new clinical criteria for AD incorporate this ideology with the designation of “pre-symptomatic AD” (Sperling et al., 2011). In the study of the complex clinicopathological spectrum of FTLD disorders, where heterogeneity is the rule, useful markers to develop homogenous clinical, genetic, and neuropathologic subgroups will be crucial to further our goals toward meaningful treatments that could potential slow disease progression and limit patient disability.

### Conflict of interest statement

Dr. John Q. Trojanowski reports single consulting services to Pfizer, J&J, MetLife, and BMS; Royalty payments through Penn licenses; and research support from AstraZeneca and BMS. The other authors declare that the research was conducted in the absence of any commercial or financial relationships that could be construed as a potential conflict of interest.

## Abbreviations

FTLD: frontotemporal lobar degeneration
AD: Alzheimer's disease
Aβ: amyloid-beta
CSF: cerebrospinal fluid
CNS: central nervous system
FTLD-tau: FTLD with tau pathology
TDP-43: TAR DNA binding protein-43
FTLD-TDP: FTLD with TDP pathology
PiD: Pick's disease
CBD: corticobasal degeneration
PSP: progressive supranuclear palsy
pathogenic *MAPT* mutations-FTDP-17: FTD and parkinsonism linked to chromosome 17
ALS: amyotrophic lateral sclerosis
FUS: fused-in-sarcoma protein
FTLD-FUS: FTLD with FUS pathology
FTLD-UPS: FTLD with tau- and TDP-43-negative ubiquitinated inclusions
FTLD-ni: FTLD in the absence of significant neuropathological inclusions
*GRN*: progranulin gene
*MAPT*: tau gene
*C9orf72*: C9orf72 gene
*VCP*: valosin-containing protein gene
*TARDBP*: TDP-43 gene
FTLD-ALS: clinical FTLD with ALS
*CHMP2B*: charged mutlivesciular body protein 2B gene
bvFTD: behavioral-variant frontotemporal dementia
PPA: primary progressive aphasia
lvPPA: logopenic-variant PPA
svPPA: semantic-variant PPA
naPPA: non-fluent aggramatic variant PPA
CBS: corticobasal syndrome
Aβ_1−42_: β-amyloid
MCI: mild cognitive impairment
t-tau: total-tau
p-tau: phosphorylated-tau
p-tau_181_: phosphorylated tau at serine 181
p-tau_231_: phosphorylated tau at threonine 231
ELISA: enzyme-linked immunosorbent assay
xMAP: luminex flow immunoassay
MRI: magnetic resonance imaging
DIAN: dominantly-inherited AD network
MTBD: microtubule-binding domain
DTI: diffusion-tensor imaging
GM: gray matter
GWAS: genome-wide association studies.
